# What Is the Best Method for Diagnosing Osteosarcopenic Adiposity in Women After Long-Term Bariatric Surgery? A Comparison and Validation of Different Criteria

**DOI:** 10.3390/nu16223965

**Published:** 2024-11-20

**Authors:** Maísa Miranda Araújo, Ricardo Moreno Lima, Kênia Mara Baiocchi de Carvalho, Patrícia Borges Botelho

**Affiliations:** 1 Graduate Program in Human Nutrition, University of Brasilia, Brasília 70910-900, Brazil; ricardomoreno@unb.br (R.M.L.); kenia@unb.br (K.M.B.d.C.); pbotelho@unicamp.br (P.B.B.); 2 Graduate Program in Physical Education, University of Brasilia, Brasília 70910-9000, Brazil; 3 Faculty of Applied Sciences, State University of Campinas, Campinas 13484-350, Brazil

**Keywords:** osteosarcopenic adiposity, osteosarcopenic obesity, sarcopenic obesity, osteoporosis, bariatric surgery, validation study

## Abstract

Background/Objectives: To evaluate the agreement and discriminant validation of different osteosarcopenic adiposity (OSAd) diagnostic criteria in women post-Roux-en-Y gastric bypass (RYGB) surgery. Methods: Surgery. This is a cross-sectional study with women ≥2 years post-RYGB. OSAd was diagnosed using three criteria: Kelly for OSAd; ESPEN/EASO and SDOC for SO, associated with WHO osteopenia, respectively. Agreement was assessed with Cohen’s Kappa, and the predictive discriminatory capability was evaluated by sensitivity, specificity, and accuracy, using impairment of physical function and the increased risk of fracture as reference standards.; Results: A total of 178 women were evaluated, with a mean age of 45.2 ± 9.6 years old and postoperative time of 6.6 ± 3.6 years. The prevalence of OSAd was 2.2% [Kelly]; 2.8% [ESPEN/EASO + WHO]; 6.2% [SDOC + WHO]. Moderate agreement was found between Kelly and ESPEN/EASO (k = 0.658, *p* < 0.001), but agreement with SDOC was null (k = 0.104, *p* = 0.114). All criteria demonstrated high specificity (94.0–98.2%) and low sensitivity (0.0–16.7%), with Kelly showing the highest accuracy (92.7%); Conclusions: Among the evaluated criteria, Kelly presented the highest accuracy and 2.2% of OSAd prevalence. Despite consistently high specificity, all criteria exhibited low sensitivity. These findings highlight the need for more sensitive diagnostic approaches for OSAd in postoperative RYGB populations.

## 1. Introduction

Sarcopenia is a progressive and generalized skeletal muscle mass disorder, characterized by loss of muscle strength, accompanied by a decline in muscle quantity and/or quality [1]. Sarcopenia can coexist with obesity and with osteopenia being termed osteosarcopenic adiposity (OSAd), originally conceptualized as osteosarcopenic obesity [2]. OSAd represents a multifaceted interplay between adipose tissue, bone, and muscle health [3], which can lead to poor physical function, higher risk of frailty and fractures, and early death as compared to their isolated components [4,5].

Despite this multifactorial disorder being more frequent in the older population, individuals who have undergone metabolic bariatric surgery (MBS) may be at an increased risk for this condition [6]. MBS is a well-known treatment for obesity, which leads to clinical and metabolic benefits; however, patients experience significant and rapid weight loss, leading to loss of muscle and bone mass and a higher risk for nutritional deficiencies, such as vitamin D and calcium [7,8]. Furthermore, there is a risk of weight regain after surgery, which depending on the magnitude may contribute to impairment of body composition and the recurrence of comorbidities [9]. Therefore, these complications as the risk of OSAd can affect individuals who have undergone MBS, especially among those who lost the multidisciplinary follow-up, typically observed after the second year after surgery [10].

So far, there is no standardized diagnostic criterion for OSAd in patients who have undergone MBS. A revised criterion was proposed by Kelly et al. (2019) including the presence of four components for adults and the elderly: lower bone mineral density (BMD), high-fat mass, low appendicular lean mass (ALM), and the presence of central obesity [3]. However, according to these criteria, the impairment of the strength was not considered for sarcopenia assessment. On the contrary, the European Society for Clinical Nutrition and Metabolism (ESPEN) and the European Association for the Study of Obesity (EASO) proposed sarcopenic obesity (SO) diagnostic criterion that include the decline of absolute muscle strength in addition to the loss of muscle mass for sarcopenia determination [11]. This contrast prompts the question: Does the presence or absence of low muscle strength in OSAd diagnosis affect its prevalence rate and/or the prediction of adverse clinical outcomes? Previously, a comparison of ESPEN/EASO consensus with other diagnosis criteria for SO among adults mid to long-term post-MBS showed a high but variable prevalence of SO (7.9–23.0%) with little agreement among the diagnostic criteria evaluated [12]. However, as far as we know, no study has assessed the prevalence of OSAd in this specific population or compared the diagnostic criteria available in the literature.

Hence, this study aims to assess the OSAd diagnostic criteria’s concurrent and discriminant validity, focusing on patients who have undergone MBS. Primarily, the following three diagnostic criteria were used to determine OSA: (1) criterion proposed by Kelly et al. (2019) [3]; (2) the SO consensus by ESPEN/EASO (2022) associated with osteopenia criterion proposed by WHO (1994) [11,13]; (3) the Sarcopenia Definition and Outcomes Consortium (SDOC) (2020) associated with osteopenia criterion proposed by WHO (1994) [13,14]. This study will contribute to enhancing the precision of OSAd diagnosis, allowing for more accurate identification, prevention, and treatment of this condition in women who have undergone MBS.

## 2. Materials and Methods

### 2.1. Study Design and Setting

This is a cross-sectional analysis of two Brazilian bariatric surgery research protocols: The CINTO cross-sectional study (Food consumption, lifestyle, control of comorbidities, and nutritional status of bariatric surgery patients study) [15], and baseline data from the NERON clinical trial study (online multicomponent intervention, based on the Brazilian Cardioprotective Diet and a resistance training program, to control metabolic parameters in people in the late postoperative period of bariatric surgery: randomized clinical trial) (registered with the Brazilian Registry of Clinical Trial under the number: RBR-4pdv53d). Both studies were approved by the Brazilian Research Ethics Committee and all participants signed an informed consent form. Recruitment of participants was conducted through an open call on social media and public and private MBS clinics. Data collection occurred from July 2019 to February 2020 and August 2023 to March 2024 at the University of Brasilia, Brasília, Brazil. Both studies adhere to the same eligibility criteria, with all parameters measured using the same equipment and following identical protocols. This study was reported following the Strengthening the Reporting of Observational Studies in Epidemiology (STROBE) checklist (Supplementary Material).

### 2.2. Participants

Adults (19–59 years old) of both sexes who had undergone roux-Y gastric bypass (RYGB) surgery for two or more years, and who had body composition and physical performance test data were included in the research protocols. This post-surgery time range was chosen because, after 2 years of surgery, patients usually reach weight stabilization, and some weight regain degree may occur [16]. Pregnant or lactating women and individuals with psychiatric conditions or using psychotropic or anti-obesity medication or with a pacemaker were excluded. For this study, male participants were excluded due to the non-representative number in the samples (*n* = 15) (Supplementary Figure S1).

### 2.3. Clinical and Socio-Demographic Data

Clinical and sociodemographic data were assessed with a semi-structured questionnaire regarding age, sex, education level, surgery date, preoperative BMI, minimum and maximum weight achieved after surgery and the presence of current comorbidities.

### 2.4. Anthropometric, Body Composition, and Bone Measurements

Body weight and height were measured by trained dietitians. Current body mass index (BMI) (kg/m^2^) was calculated [17]. Total weight loss (kg and %) and excess weight loss (%EWL) were calculated using a BMI of 25 kg/m^2^ as the ideal body weight [16]. Weight regain was calculated using the percentage of maximum weight, i.e., [100 × (current weight − nadir weight)]/(preoperative weight − nadir weight). Significative weight regain (%) was considered when values were ≥20%.

Body composition and bone parameters were evaluated using dual X-ray absorptiometry (DXA) (GE Lunar DPX-IQ, Madison, WI, USA). Participants were orientated to wear light clothing and be barefoot for the exam. Body fat mass (%), skeletal muscle mass (SMM, kg), appendicular lean mass (ALM, kg), android to gynoid fat ratio, and hip axis length were used. To assess osteoporosis/osteopenia, the bone mineral density (BMD) and t-score and z-score from the lumbar spine (L1–L4) and femoral neck were assessed for post-menopausal women and pre- and peri-menopausal women, respectively. The risk of fracture was estimated by the hip axis length ≥ 98.73 mm, as proposed by Ha et al. 2019 for predicting the occurrence of hip fracture [18].

### 2.5. Strength and Physical Function Parameters

Muscle strength was evaluated by handgrip strength, 30 s chair test (30-CST), and 5 times sit-to-stand test (5STS). Trained evaluators performed all tests. For handgrip strength, participants were instructed to sit with arms at 90°, the forearm in a neutral position, and the wrist between 0 to 30° of extension [19]. At the evaluator’s verbal command, participants pressed the dynamometer (JAMAR^®^, Columbia, MD, USA) as tightly as possible. Three attempts of each hand were performed with an interval of 60 s rest between them. Maximum strength between the two hands was used for determining the HSG, as recommended in ESPEN/EASO (2022) SO consensus [11]. For 5STS, participants were seated in a standard-height chair with arms crossed on the chest, and they were oriented to stand up and sit down completely as quickly as possible five times [20]. Two attempts were made, and the lowest time was used. For the 30-CTS [21], participants were instructed to sit the same way as the 5STS, and complete as many times as possible within 30 s. Only complete movements were recorded [22].

Impaired physical function was defined by altered time up and Go (TUG) and 6 min walk test (6MWT). For TUG, participants begin the test sitting in a standard-height chair, with their back against the chair, both arms resting on the things, and feet on the ground. At the evaluator’s signal, they were instructed to get up and walk forward, without running and turn around a cone, and return to the chair, as fast as possible, completing 3 m distance [23]. Three attempts were made, with a 60 s recovery time. The lowest time was used. Altered TUG was defined according to the Furlanetto et al. (2022) cut-off point for Brazilian adults [24]. For 6MWT, at the evaluator’s command, participants walked as fast as possible, without running, for 6 min around 50 yards (45.7 m) in length marked by cones. For this test, one attempt was made, and it was considered the total number of meters covered by the participants [22]. Altered 6MWT was defined by Enrichi et al. (1998) for adults [25].

### 2.6. Osteosarcopenic Adiposity (OSAd) Criteria

Primarily, two diagnostic criteria were used to define OSAd. First, we applied the OSA-specific criterion proposed by Kelly et al. (2019) [3] (Supplementary Table S1). For comparison, we used the SO consensus developed by ESPEN/EASO and the sarcopenia consensus developed by SDOC, associated with WHO criteria for osteopenia/osteoporosis diagnosis [11,13,14]. The cutoff points used followed those proposed by each criterion. For SO by ESPEN/EASO criterion, cutoff points developed for Brazilian adults, when available, were used as recommended in this consensus [11] (Supplementary Table S1). The SDOC criterion do not propose a definition of obesity and osteopenia/osteoporosis, therefore, for this OSAd criterion, we uniformly used the definition endorsed by ESPEN/EASO SO consensus recommendations for obesity and the WHO criterion for osteopenia/osteoporosis diagnosis [11,13,14].

### 2.7. Statistical Analysis

The Kolmogorov–Smirnov test was performed to evaluate the normality of the data distribution. Categorical variables were presented as frequency (%, *n*), and continuous variables as mean or median and standard deviation or interquartile range.

To determine the OSAd identification agreement between the diagnostic criteria, Cohen’s Kappa was calculated, as it evaluates binary scales (presence and absence) and accounts for marginal heterogeneity in the data [26]. The Kappa coefficient was interpreted according to McHugh’s (2012) proposed classification [27].

To evaluate the predictive discriminatory capability of different OSAd criteria, we used the impairment of physical function by TUG and 6MWD, and the risk of fracture by hip axis length as reference standards. Sensitivity, specificity, positive predictive value (PPV), negative predictive value (NPV), and their respective 95% confidence intervals (95% CI) were calculated for the three criteria assessed: Kelly et al., (2019), ESPEN/EASO (2022), and SDOC (2020) [3,11,14].

Data were analyzed using IBM SPSS Statistics for IOS, version 26 (IBM Corp., Armonk, NY, USA).

## 3. Results

### 3.1. Population Characteristics

A total of 178 women were evaluated. The mean age of the participants was 45.20 ± 9.59 years old, with 6.63 ± 3.61 years of postoperative time. In total, 53.9% of the women were postmenopausal (Table 1). Participants’ %EWL was 68.53 ± 27.31% with a current BMI of 31.05 ± 5.70 kg/m^2^. Significant weight regain was seen in 85.4% of the sample.

Using Kelly et al.’s (2019) [3] cut-off points, 97.2% presented excess body fat mass and 22.5% predominant central adiposity; 77.0% low muscle mass, assessed by adjusted-height ALM (Table 2). Using the ESPEN/EASO cut-off point, 75.3% had excess body fat mass and 67.4% low muscle mass, assessed by ALM adjusted by weight percentage. Only two women (1.1%) presented low absolute handgrip strength. This number increased when handgrip strength was adjusted by BMI (*n* = 45, 25.3%), fat mass (*n* = 52, 29.2%), and body weight (*n* = 63, 37.1%). Low 5STS and 30-CST were observed in 11.8% and 15.2% of the sample, respectively.

**Figure 1:**
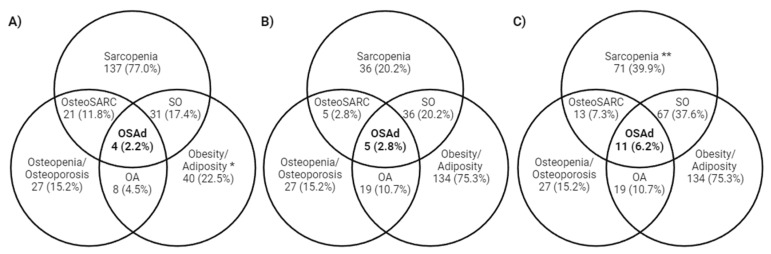
Venn diagram of the parameters evaluated by Kelly et al. (2019) [3] for osteosarcopenic adiposity (**A**); ESPEN/EASO sarcopenic obesity + WHO osteopenia/osteoporosis (**B**); and SDOC sarcopenic obesity + WHO osteopenia/osteoporosis (**C**) criteria for diagnosing OSAd in women who have undergone bariatric surgery. * The diagnosis of adiposity diagnosis was determined by the excess of body fat mass and the presence of central obesity, according to Kelly et al.’s (2019) [3] criteria. ** The diagnosis of sarcopenia was determined by low muscle relative strength, according to the SDOC criterion. Abbreviations: OsteoSARC: osteosarcopenia; SO: sarcopenic obesity; OA: osteopenic adiposity; EASO: European Association for the Study of Obesity; ESPEN: European Society for Clinical Nutritional and Metabolism; SDOC: Sarcopenia Definitions and Outcomes Consortium; WHO: World Health Organization.

Overall, applying the criterion recommended by Kelly et al. (2019) [3], only 22.5% of the participants were diagnosed with adiposity (indicating an excess of %fat mass and the presence of central adiposity distribution), while most of the sample was diagnosed with sarcopenia (77.0%). The combination of these two outcomes, which is sarcopenic obesity was observed in 17.4%. Although the ESPEN/EASO (2022) [11] criterion identified a high prevalence of obesity (75.3%), the rates of sarcopenia (20.2%) and SO (20.2%) were lower compared to earlier criteria, which only assessed low appendicular lean mass (ALM) adjusted for height, without considering muscle strength. The prevalence of sarcopenia (39.9%) and SO (37.6%) using the relative strength, according to the SDOC (2020) [14] cut-off point, rather than absolute strength, was higher than both cited criteria. Osteopenia/osteoporosis was equally prevalent in both criteria (15.2%), determined by the same femoral neck or lumbar spine cut-off point. Notably, the prevalence of OSAd was higher when using the SDOC (2020) [14] cut-off (6.2%), compared to Kelly et al. (2019) [3] (2.2%) and ESPEN/EASO (2.8%) (see Figure 1).

### 3.2. Osteosarcopenic Adiposity (OSA) Validation

When evaluating the content validity between the OSAd prevalence using Kelly et al. (2019) [3] versus the ESPEN/EASO (2022) [11] criterion, Cohen’s Kappa test revealed a moderate agreement between the two diagnosis criteria (k = 0.658; *p* < 0.001). No agreement was observed between Kelly et al. (2019) [3] versus SDOC (2020) [14] + WHO (1994) [13] (k = 0.104; *p* = 0.114). (Table 3).

Across all criteria, sensitivity was low (0.0–16.7%), indicating poor detection of true OSAd cases (Table 4). Specificity was high for all criteria, particularly in the TUG and 6MWT tests (up to 98.2% for Kelly et al. [3] and 96.5% for Kelly et al. [3] in 6MWT), suggesting a robust capability to correctly exclude non-OSAd individuals. Accuracy, which estimates the proportions of all correct predictions (true positives and true negatives) of all predictions, was highest for Kelly et al. [3] in the TUG (92.7%) and 6MWT (91.4%) tests. All criteria demonstrated low accuracy when using the hip axis length test as the gold standard for diagnosing OSAd. Positive predictive values (PPV) were uniformly low to medium (0.0–66.7%), reflecting a moderate probability of having OSAd given a positive test result. Negative predictive values (NPV) were high in TUG and 6MWT tests (up to 94.4% for Kelly et al. [3]), indicating a strong ability to rule out OSAd when results were negative (Table 4).

**Figure 2:**
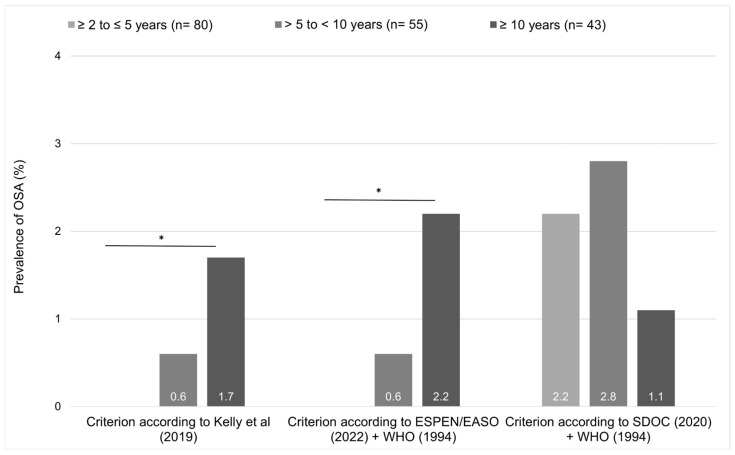
Osteosarcopenic adiposity (OSAd) prevalence according to diagnostic criteria and postoperative surgical timepoints. * *p*-value < 0.05. Abbreviations: EASO: European Association for the Study of Obesity; ESPEN: European Society for Clinical Nutritional and Metabolism; OSA: osteosarcopenic adiposity; SDOC: Sarcopenia Definitions and Outcomes Consortium; WHO: World Health Organization. References cited in the figure: Kelly et al. (2019) [3]; ESPEN/EASO (2022) [11] + WHO (1994) [13]; SDOC (2020) [14] + WHO (1994) [13].

The prevalence of OSAd appears to be associated with longer post-MBS time (Figure 2). The prevalence of OSAd according to both Kelly et al. (2019) [3] and ESPEN + WHO OSAd diagnostic significantly increased among the postoperative time points [ESPEN/EASO (2022) [11] + WHO (1994) [13]: X^2^(2) = 13.725; *p* < 0.002; Kelly et al. (2019) [3]: X^2^(2) = 9.401; *p* = 0.009]. However, with SDOC (2020) [14] + WHO (1994) [13] diagnostic criterion, this association was not observed [X^2^(2) = 0.414; *p* = 0.917].

### 3.3. Osteosarcopenic Adiposity (OSA) and Its Association with Postoperative Time and Weight Regain

The group with OSAd presented a higher median weight regain (%) (44.1%) compared to the group without OSAd (28.9%, *p* = 0.033). This association was not observed with ESPEN/EASO + WHO and SDOC + WHO criteria (Supplementary Table S2).

## 4. Discussion

This is the first study to evaluate the prevalence of OSAd using different criteria in women in late postoperative MBS. SDOC (2020) [14] consensus + WHO (1994) [13] shows a higher prevalence of OSAd, however with a null and minimal agreement respectively between Kelly et al. (2019) [3] and ESPEN/EASO (2022) [11] + WHO (1994) [13]. Regarding the Kelly et al. (2019) [3] and ESPEN/EASO (2022) [11] + WHO (1994) [13] criteria, those identified a similar prevalence of OSAd rate with a moderate agreement between them [2,11,13]. Our findings revealed a consistently high specificity across all criteria and tests, and a low sensitivity was observed across all criteria. Among the criteria evaluated, Kelly et al. (2019) demonstrated the highest overall accuracy and NPV, particularly in the TUG and 6MWT tests, making it more reliable for diagnosing OSAd in women post-bariatric surgery, compared to the others [3].

The OSAd prevalence among women who underwent MBS found in our study (2.2–6.2%) was relatively lower than the global estimated prevalence of 8% (95% CI: 6–11%) in middle-aged and older adults (ranging from 58.1 to 81 years old) [6]. The prevalence of OSAd further increases to 13% in older individuals (≥60 years) [6]. This difference could be attributed to the wide range of definitions used to define this syndrome in the evaluated studies and also to the younger age of our population since age is a major factor in the development of OSAd [2].

Despite the low prevalence of OSAd, we observed a high prevalence of sarcopenic obesity (17.4% to 37.6%), aligning with the findings of Buzza et al. (2020), who reported a 28.3% prevalence of sarcopenia in women with obesity who had undergone RGYB for at least 2 years [28]. Notably, a substantial difference can be seen in the sarcopenia and obesity prevalence across the diagnostic criteria evaluated, which can be attributed to the differing methods applied to assess those components, for instance, the consideration solely of low muscle mass in sarcopenic definition by Kelly et al. (2019), while ESPEN/EASO also considered low absolute strength [3,11]. The reliance on muscle mass alone in the Kelly criteria has been associated with higher rates of sarcopenia diagnosis (77%) compared to the 20.2% and 39.95% observed with ESPEN and SDOC, respectively.

Muscle strength is a well-recognized marker of physical health; its decline is associated with higher rates of morbidity, disability, and mortality [29,30]. The incorporation of low muscle strength in sarcopenic diagnosis is due to its robust predictive capability of adverse clinical outcomes, compared to the isolated muscle mass (pre-sarcopenia) [1]. As result, international societies and the scientific community have recognized muscle strength as an essential component in the diagnostic criteria for sarcopenia [1,11,14]. Furthermore, it also predicts difficulty in daily tasks, leading to a loss of autonomy [31]. In our study, only two participants showed low absolute handgrip strength. However, when the handgrip strength was adjusted for body mass and fat mass a higher prevalence of low muscle strength was found. This is important because absolute muscle strength has limitations in obese individuals, who typically show higher values due to larger body size [32]. Nevertheless, increased intramuscular fat can disrupt force transmission and impair motor unit recruitment, reducing force production and necessitating even higher absolute strength levels for equivalent tasks [33]. This highlights the importance of considering muscle strength assessment and adjust it for body size to diagnose sarcopenia in individuals with obesity.

However, the SDOC + WHO criteria, which utilize relative strength, showed low sensitivity and accuracy, while the Kelly et al. (2019) criteria, which consider only muscle mass, demonstrated higher sensibility and accuracy [3,13,14]. One possible explanation may be lie not in the assessment of muscle strength, but in the significant differences in how they determine obesity. Kelly et al. (2019) [3] consider alongside the excess of fat mass the presence of central adiposity, while the other two criteria only consider overall fat mass. This methodological difference resulted in a discrepancy in obesity/adiposity prevalence rates (Kelly et al. report a 22.5%, in contrast to 75.3% found in the ESPEN and SDOC criteria). Initially, the proposed criteria for OSAd in 2014 did not include central obesity [2]. However, this type of fat distribution is closely linked to poorer health outcomes [3]. In Perna and colleagues’ studies, OSAd was divided into two types based on fat distribution: central predominant and subcutaneous predominant [34]. Among 801 hospital patients (≥65 years), the prevalence of OSAd was 6.79% [34]. The central type was more prevalent (5.56%) compared to the subcutaneous type (2.22%). The central type was associated with worse metabolic health, inflammation profile, and risk of fractures [34], as a result of systemic inflammation promoted by an increase of adipose tissue-derived cytokines (resistin, TNF-α, and IL-6), which accelerate bone resorption and suppress bone formation [35]. Additionally, it disrupts bone remodeling by increasing sympathetic activity, thereby reducing osteoblast function and increasing osteoclast activity, ultimately weakening bone structure and increasing fracture susceptibility [36]. These factors weaken bone structure and increase susceptibility to fractures [37]. These findings suggest that central obesity may be frequent in OSAd and favor more adverse outcomes. Thus, considering central obesity in OSAd assessment should be encouraged for accurately identifying the patients with this condition, as well as the relative muscle strength.

Despite the higher accuracy of the Kelly et al. [3] criterion, a significantly low sensitivity was found in all evaluated criteria, ranging from 0.0% to 16.7%. This low sensitivity suggests a weak ability to identify true OSAd cases, which is a critical concern for clinical practice as it indicates that many true OSAd cases might go undetected. One plausible reason for this could be that the population evaluated may primarily be in the early stages of the disease and may not yet manifest significant physical impairments or increased fracture risk detectable by hip axis length or physical performance tests such as TUG and 5-MWT, leading to underdiagnosis when using these tests. Furthermore, the Kelly et al. [3] criterion do not incorporate muscle strength assessment, which is increasingly recognized as an important component in diagnosing OSAd. The omission of muscle strength may compromise the sensitivity of these criteria, given its strong association with mobility and physical function [29,30,31]. Additionally, the cut-offs for OSAd components such as sarcopenia by low skeletal muscle mass index in the Kelly et al. [3] criterion, and muscle strength (5STS) in the ESPEN/EASO criterion were set for general adults and elderly [3,11]. Thus, these cut-offs may not be sufficiently sensitive to detect early or mild cases of OSAd in adults after a rapid and significant weight loss, which could result in a low prevalence of OSAd found in our study. The low prevalence of OSAd possibly contributed to a lower sensitivity, as the higher the prevalence of the disease, the greater the odds of detecting true cases. Therefore, our findings suggest the need for the development of a more sensitive test or biomarker for OSAd risk and an establishment of cut-offs specific for patients who have undergone MBS to detect the early and mild onset of OSAd.

Furthermore, our study demonstrates that OSAd prevalence typically manifests after 5 years post-MBS and increases with longer postoperative time. This phenomenon may be attributed to several factors, including challenges in maintaining long-term follow-up support, adherence to medical and nutritional recommendations, the return of unhealthy habits, such as a sedentary lifestyle, and the physiological effects of aging which exacerbate the loss of muscle and bone mass [10,38]. Moreover, OSAd diagnosis, particularly when assessed using the Kelly et al. (2019) criterion, was associated with higher weight regain [3]. These insights reinforce the need for continuous follow-up to monitor and manage OSAd and its components after MBS. Data from recent studies suggests that nutrient-dense food with higher protein, calcium, and vitamin D and C, along with an active lifestyle and possibly assisted resistance training is beneficial to maintain satisfactory body composition and manage OSAd [39]. Particularly after MBS, supplementation of vitamin D and calcium is essential and recommended for these patients, as well as DXA performed in the second-year post-surgery for bone density assessments [40]. For those women in the postmenopausal stage after MBS, annual DXA assessment is advised [41].

To the best of our knowledge, this is the first study investigating OSAd prevalence in individuals who underwent MBS and comparing three OSAd diagnosis criteria available in the literature. Our study used the DXA, a gold-standard method to access body composition, and when available, the population-specific cut-off points for body composition and physical function parameters were applied (i.e., Brazilian adults). However, some limitations such as the lack of a gold standard test for OSAd diagnosis are a significant challenge that impacts the sensitivity and overall diagnostic accuracy observed. In the absence of gold-standard diagnostic criterion, this study relied on available physical performance tests and hip axis length to determine risk fracture as proxies, which may not fully capture the nuanced and early manifestations of OSAd, leading to lower diagnostic sensitivity. Additionally, the convenience sample used in our study may affect the generalizability of our findings to the broader population of women who underwent RYGB. The lack of statical power assessment also be considered, requiring caution in drawing definitive conclusions about the relative superiority of one diagnostic criterion over another. In our study, we did not perform stratified analysis accounting for comorbidities due to small sample size. The presence of comorbidities might influence the prevalence of OSAd, since the metabolic abnormalities are associated with decreased lean body mass, increased body fat and low-grade chronic inflammation, all of which can contribute to OSAd [34,35]. New research should concentrate resources on refining the OSAd diagnostic criterion, developing more sensitive and specific markers or imaging techniques that can better detect early-stage OSAd, and then improving the diagnostic accuracy and patient outcomes. Studies should use larger and representative samples, as well as considerer the impact of comorbidities in OSAd prevalence. Therefore, our study should be considered as a step toward better understanding the complexities of OSAd diagnosis in post-bariatric surgery patients and encourages future research.

## 5. Conclusions

In this study, which evaluated different OSAd diagnostic criteria in women who underwent MBS surgery, a moderate agreement was observed between the Kelly et al. [3] and ESPEN/EASO criteria, while agreement with the SDOC criterion was absent. A high specificity and low sensitivity were found in all criteria, with Kelly et al. [3] showing the highest accuracy, suggesting its potential clinical utility. However, these findings underscore the necessity for more sensitive diagnostic tools for this population. Our study also reinforces the postoperative care following MBS, since OSAd prevalence seems to increase over time post-surgery and is associated with higher weight regain. Future research should focus on refining OSAd diagnostic criterion to enhance sensitivity without compromising specificity, thereby improving the detection and management of this condition post-bariatric surgery and mitigating its potential health impacts in the long term.

## Figures and Tables

**Table 1:** Anthropometric, sociodemographic, and clinical profile of women who underwent bariatric and metabolic surgery.

Participant Characteristic (Mean ± SD or *n*; %)	Total (*n* = 178)
Age (years)	45.20 ± 9.59
Education level (years)	
0–8 years	17 (8.80)
9–11 years	67 (34.70)
12 or more years	109 (56.50)
Postmenopausal	96 (53.9)
Preoperative BMI (kg/m^2^)	42.76 ± 7.05
Postoperative years	6.63 ± 3.61
Current weight (kg)	80.52 ± 15.34
Current BMI (kg/m^2^)	31.05 ± 5.70
Total Weight Loss (kg)	30.20 ± 14.11
Total Weight Loss (%)	26.95 ± 10.47
Excess weight loss (%)	68.53 ± 27.31
Weight regain (kg)	10.20 ± 8.08
Weight regain (%)	27.30 ± 19.27
Significant weight regain ≥ 20%	109 (61.23)
Weight regain (kg), of all with significant weight regain (*n* = 109)	15.11 ± 7.70
Weight regain (%), of all with significant weight regain (*n* = 109)	39.85 ± 16.48
Comorbidities	
Pre-diabetes	6 (3.37)
Polycystic ovary syndrome	14 (7.87)
Hypertension	31 (17.42)
Cardiovascular disease	12 (6.74)
Dyslipidemia	15 (8.43)
Obstructive sleep apnea	15 (8.43)
Endometriosis	7 (3.93)
Hepatic steatosis	10 (5.62)
Asthma	8 (4.49)

**Table 2:** Body composition and physical function parameters of women who underwent bariatric surgery.

Parameters	Total (*n* = 178)
Body Composition	
Fat Mass (kg)	36.52 ± 11.61
Fat Mass (%)	45.55 ± 7.53
Excess Fat Mass (%)	
Kelly et al., 2019 [3] * (*n* (%))	173 (97.20)
ESPEN/EASO (*n* (%))	134 (75.30)
Fat mass index (kg/m^2^)	14.10 ± 4.51
A/G Fat Mass ratio	1.22 ± 0.14
Central Obesity (*n* (%))	40 (22.47)
ALM (kg)	17.30 ± 2.61
ALM/height^2^ (kg/m^2^)	6.66 ± 0.83
ALM/weight × 100 (%)	21.88 ± 3.31
SMM (kg)	40.41 ± 6.12
SMM/weight × 100 (%)	51.10 ± 7.90
Low muscle mass	
ALM/height^2^-Kelly et al., 2019 [3] (*n* (%))	137 (77.00)
ALM/weight × 100-ESPEN/EASO ** (*n* (%))	120 (67.40)
BMD lumbar spine (g/cm^2^)	1.17 ± 0.16
BMD femoral neck (g/cm^2^)	0.95 ± 0.28
Physical function	
Absolute handgrip strength (kg)	28.90 ± 5.38
Low absolute handgrip strength (*n* (%))	2 (1.1)
Handgrip strength/BMI	0.95 ± 0.22
Low handgrip strength/BMI (*n* (%))	45 (25.30)
Handgrip strength/fat mass	0.88 ± 0.35
Low handgrip strength/fat mass (*n* (%))	52 (29.20)
Handgrip strength/weight	0.37 ± 0.08
Low handgrip strength/weight (*n* (%))	66 (37.1)
5STS (s)	10.34 ± 2.36
Low 5STS (*n* (%))	21 (11.80)
30-CST (rep)	13.37 ± 4.15
Low 30-CST	27 (15.20)
TUG (minutes)	6.30 ± 1.06
Altered TUG (*n* (%))	11 (6.2)
6MWT (m)	513.63 ± 76.39
Altered 6MWT (*n* (%))	6 (3.4)

* The value presented is based on total body fat mass alone and does not represent adiposity diagnostic prevalence by the Kelly et al. (2019) [3] criterion. ** The value presented is based on total body composition alone and does not represent sarcopenic diagnostic prevalence by ESPEN/EASO consensus. Abbreviations: BMD: bone mineral density; BMI: body mass index; kg: kilograms; m: meters; rep: repetitions. 5STS: 5 times sit-to-stand test; 6MWT: 6 min walk test; 30-CST: 30 s chair test; TUG: time-up and go test.

**Table 3:** Cross classification and agreement analysis of osteosarcopenic adiposity (OSAd) prevalence between diagnostic criteria in women who underwent bariatric surgery.

Diagnostic Criteria	Diagnostic Criteria			Agreement	
				Kappa	*p*-Value
	ESPEN/EASO (2022) [11] + WHO (1994) Osteoporosis/Osteopenia [13]				
		−OSAd, *n* (%)	+OSAd, *n* (%)		
OSAd (Kelly et al.) [3]	−OSAd, *n* (%)	172 (98.9)	2 (1.1)	0.658	<0.001
	+OSAd, *n* (%)	1 (25.0)	3 (75.0)		
	SDOC (2022) [14] + WHO (1994) osteoporosis/osteopenia [13]				
OSAd (Kelly et al.) [3]		−OSAd, *n* (%)	+OSAd, *n* (%)		
	−OSAd, *n* (%)	164 (94.3)	10 (5.7)	0.104	0.114
	+OSAd, *n* (%)	3 (75.0)	1 (25.0)		
	ESPEN/EASO (2022) [11] + WHO (1994) osteoporosis/osteopenia [13]				
SDOC (2020) [14] + WHO (1994) osteoporosis/osteopenia [13]		−OSAd, *n* (%)	+OSAd, *n* (%)		
	−OSAd, *n* (%)	165 (95.4)	8 (4.6)	0.350	<0.001
	+OSAd, *n (*%)	2 (40.0)	3 (60.0)		

Abbreviations: EASO: European Association for the Study of Obesity; ESPEN: European Society for Clinical Nutritional and Metabolism; OSA: osteosarcopenic adiposity; SDOC: Sarcopenia Definitions and Outcomes Consortium; WHO: World Health Organization.

**Table 4:** Sensitivity, specificity, PPV, NPV for the validation of different osteosarcopenic adiposity (OSAd) diagnostic criteria according to impairment of physical performance test in women who underwent bariatric surgery.

Diagnostic Criteria *	Sensibility (%)	Specificity (%)	Accuracy (%)	PPV (%)	NPV (%)
Time-up and Go (TUG)					
Kelly et al. (2019) [3]	9.1 (1.0, 34.3)	98.2 (95.4, 99.5)	92.7 (88.2, 95.9)	25.00 (1.6, 72.2)	94.2 (90.1, 97.1)
ESPEN/EASO (2022) [11] + WHO (1994) [13]	9.1 (1.0, 34.3)	97.6 (94.5, 99.2)	92.1 (87.5, 95.5)	20.00 (1.6, 72.2)	94.2 (90.1, 97.1)
SDOC (2020) [14] + WHO (1994) [13]	9.1 (1.0, 34.3)	94.0 (89.7, 96.9)	88.7 (83.5, 92.8)	9.09 (1.0, 34.3)	94.0 (89.7, 96.9)
6-min Walking test (6MWT)					
Kelly et al. (2019) [3]	16.7 (0.0, 0.5)	96.5 (0.9, 0.9)	91.4 (84.6, 96.0)	25.0 (1.6, 72.2)	94.4 (88.3, 97.9)
ESPEN/EASO (2022) [11] + WHO (1994) [13]	16.7 (0.0, 0.5)	95.4 (0.9, 0.9)	90.3 (83.2, 95.2)	20.0 (1.3, 62.8)	94.3 (88.2, 97.9)
SDOC (2020) [14] + WHO (1994) [13]	0.0 (-)	95.4 (89.6, 98.5)	89.2 (81.9, 94.5)	0.0 (-)	93.3 (86.8, 97.3)
Hip axis length (HAL)					
Kelly et al. (2019) [3]	1.0 (0.1, 0.4)	96.1 (8.8, 9.9)	32.5 (2.5, 4.0)	33.3 (2.3, 8.4)	32.4 (2.5, 4.0)
ESPEN/EASO (2022) [11] + WHO (1994) [13]	1.9 (0.3, 0.6)	96.1 (8.8, 9.9)	33.1 (2.6, 4.1)	33.1 (1.1, 8.9)	50.0 (2.5, 4.0)
SDOC (2020) [14] + WHO (1994) [13]	5.8 (0.2, 1.1)	94.1 (8.5, 9.8)	35.1 (2.8, 4.3)	66.7 (3.4, 9.0)	33.1 (2.6, 4.1)

* Data presented in % (95%CI).

## Data Availability

Data described in the manuscript, including the code book and analytic code, are not publicly available due to privacy restrictions but can available upon reasonable request to the corresponding author.

## Supplementary Materials

The following supporting information can be downloaded at: https://www.mdpi.com/article/10.3390/nu16223965/s1, Figure S1: Flowchart diagram of included participants. Table S1: Osteosarcopenic adiposity (OSA) classification and cut-off. Table S2: Comparison of weight regain (%) between the groups with and without OSAd presence. References [41,42,43,44,45,46,47] are cited in the Supplementary Materials.
