# Supplementary material for: What Is the Best Method for Diagnosing Osteosarcopenic Adiposity in Women After Long-Term Bariatric Surgery? A Comparison and Validation of Different Criteria
Source: Nutrients. 2024 Nov 20;16(22):3965. doi: 10.3390/nu16223965 (PMC11597721; doi:10.3390/nu16223965)
Supplement: Supplementary file 1 [file nutrients-16-03965-s001.zip › nutrients-3227945-supplementary.pdf]

Supplementary Material

Supplementary Figure S1. Flowchart diagram of included participants.

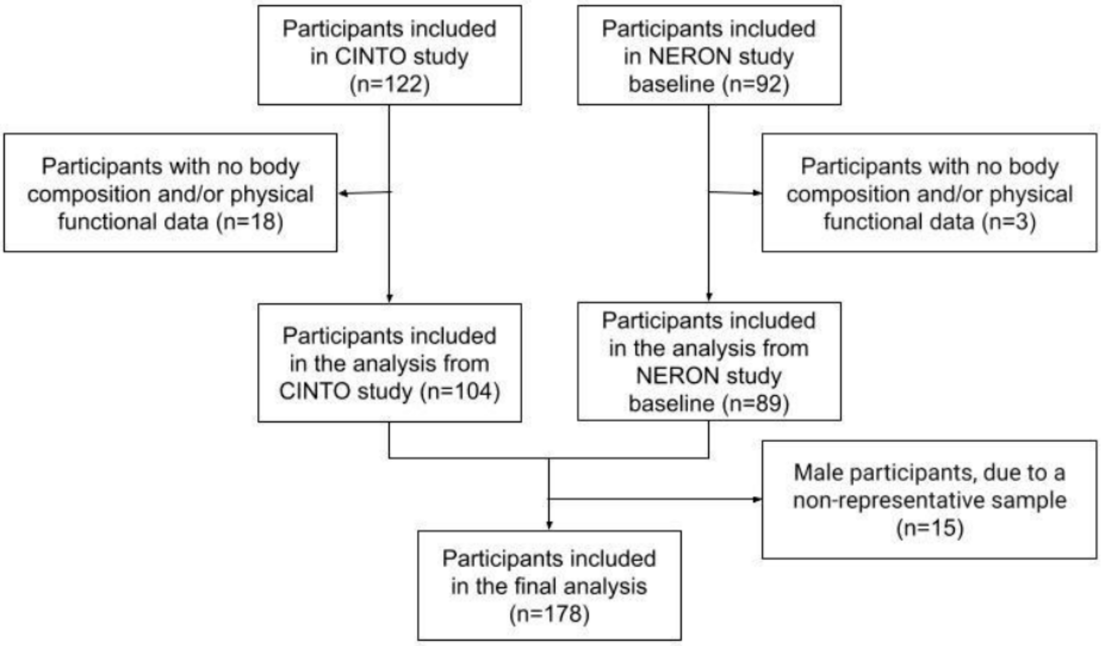

Supplementary Table S1. Osteosarcopenic adiposity (OSAd) classification and cut-off.

OSAd Criteria	Sarcopenia	Obesity/Adiposity	Osteopenia/Osteoporosis
Kelly *et al*., 2019 [1]	SMI index (ALM/height^2) [2]:<br>≤ 7.26 kg/m^2 ♀.	%Fat mass [3]:<br>≥ 32% ♀;<br>**OR**<br>Fat mass index [4]<br>≥ 13 kg/m^2 ♀;<br>**AND**<br>*Central adiposity*<br>A/G ratio > 1.0 [5].	*Pós-menopausal:*<br>t-score for BMD at the femoral neck or lumbar spine ≤ -1.0 [6].<br>**OR**<br>*Pré and peri-menopausal:*<br>z-score for BMD at the femoral neck or lumbar spine ≤ -2.0 [6].
SO ESPEN/EASO, consensus [8]<br>+<br>osteopenia/osteoporosis by WHO, 1994 criteria [6]	ALM/weight x 100, 18-65y [8]:<br>< 23.47% ♀;<br>**AND**<br>*Low muscle strength*<br>handgrip strength Brazilian [9]:<br>< 16 kg ♀;	%Fat mas [12]:<br>18-39 y:<br>≥ 39% ♀;<br>40-59 y:<br>≥ 41% ♀.	*Pós-menopausal:*<br>t-score for BMD at the femoral neck or lumbar spine ≤ -1.0 [6].<br>**OR**<br>*Pré and peri-menopausal:*

	**OR**<br>5STS, Brazilian [10]<br>> 11.64s<br>**OR**<br>30-CST, Brazilian [11]<br>20-29y: <15rep ♀;<br>30-39y: <13rep ♀;<br>40-49y: <13rep ♀;<br>50-59y: <11rep ♀;<br>60-69y: <11rep ♀.		z-score for BMD at the femoral neck or lumbar spine ≤ -2.0 [6].
SDOC consensus [13]<br>+<br>osteopenia/osteoporosis by WHO, 1994 criteria [6]	*Low muscle strength*<br>handgrip strength/BMI [13]:<br><0.79 kg/m^2 ♀;<br>**OR**<br>handgrip strength/Fat mass [13]:<br><0.65 kg/kg ♀;<br>**OR**<br>handgrip strength/weight [13]:<br><0.34 kg/kg ♀.	%Fat mass [12]:<br>18-39 y:<br>≥ 39% ♀;<br>40-59 y:<br>≥ 41% ♀.	*Pós-menopausal:*<br>t-score for BMD at the femoral neck or lumbar spine ≤ -1.0 [6].<br><br>**OR**<br>*Pré and peri-menopausal:*<br>z-score for BMD at the femoral neck or lumbar spine ≤ -2.0 [6].

Abbreviations: ALM: Appendicular Lean Mass; A/G ratio: Android Gynoide ratio; BMD: Bone Mineral Density; SMI: Skeletal Muss Index; s: seconds; a: years; rep: repetitions; EASO: European Association for the Study of Obesity; ESPEN: European Society for Clinical Nutrition and Metabolism; SMI: Skeletal muscle mass index; WHO: World Health Organization. 5STS: Five times sit and stand test; 30-CST: 30 seconds chair stand test; SDOC: Sarcopenia Definitions and Outcomes Consortium.

Supplementary Table S2. Comparison of weight regain (%) between the groups with and without OSAd presence.

Diagnostic criteria	Weight Regain (%)		
	**OSAd -**	**OSAd +**	**p-value**
Kelly et al (2019)	26.9 (14.6,38.5)	44.1 (34.7,58.4)	**0.033**
ESPEN/EASON (2022) + WHO (1994)	27.3 (14.8, 39.0)	32.6 (16.8,54,6)	0.112
SDOC (2020) + WHO (1994)	27.3 (14.2,39.1)	28.2 (19.9,42.2)	0.417

Mann-Whitney test. Data presented as median (percentile 25th - percentile 75th).

Abbreviations: EASO: European Association for the Study of Obesity; ESPEN: European Society for Clinical Nutritional and Metabolism; OSAd: Osteosarcopenic Adiposity; SDOC: Sarcopenia Definitions and Outcomes Consortium; WHO: World Health Organization.

Reference

1. Kelly, O.J.; Gilman, J.C.; Boschiero, D.; Ilich, J.Z. Osteosarcopenic Obesity: Current Knowledge, Revised Identification Criteria and Treatment Principles. *Nutrients* 2019, **11**(4), 747. https://doi.org/10.3390/nu11040747.
2. Stoklossa, C.A.J.; Sharma, A.M., Forhan, M.; Siervo, M.; Padwal, R.S.; Prado, C.M. Prevalence of sarcopenic obesity in adults with class II/III obesity using different diagnostic criteria. *J. Nutr. Metab.* 2017, **2017**, 7307618. https://doi.org/10.1155/2017/7307618.
3. American Council on Exercise. Percent Body Fat Norms for Men and Women. [(Accessed on 9 February 2024)]; Available online: https://www.acefitness.org/education-and.
4. Kelly, T.L.; Wilson, K.E.; Heymsfield, S.B. Dual Energy X-Ray Absorptiometry Body Composition Reference Values from NHANES. *PLoS ONE* 2009, **4**(9), e7038. https://doi.org/10.1371/journal.pone.0007038.
5. Imboden, M.T.; Welch, W.A.; Swartz, A.M. et al. Reference Standards for Body Fat Measures Using GE Dual Energy X-Ray Absorptiometry in Caucasian Adults. *PLoS ONE* 2017, **12**(4), e0175110. https://doi.org/10.1371/journal.pone.0175110.
6. Kanis, J.A. Assessment of Fracture Risk and Its Application to Screening for Postmenopausal Osteoporosis: Synopsis of a WHO Report. *WHO Study Group Osteoporos. Int.* 1994, **4**(6), 368–381.
7. Donini, L.M.; Busetto, L.; Bischoff, S.C.; Cederholm, T.; Ballesteros-Pomar, M.D.; Batsis, J. Definition and Diagnostic Criteria for Sarcopenic Obesity: ESPEN and EASO Consensus Statement. *Obes. Facts* 2022, **15**(3), 321-335. https://doi.org/10.1159/000521241.
8. Poggiogalle, E.; Lubrano, C.; Sergi, G.; Coin, A.; Gnessi, L.; Mariani, S. et al. Sarcopenic Obesity and Metabolic Syndrome in Adult Caucasian Subjects. *J. Nutr. Health Aging* 2016, **20**(9), 958-963.
9. Bielemann, R.M.; Gigante, D.P.; Horta, B.L. Birth Weight, Intrauterine Growth Restriction and Nutritional Status in Childhood in Relation to Grip Strength in Adults: From the 1982 Pelotas (Brazil) Birth Cohort. *Nutrition* 2016, **32**(2), 228–234.
10. Porto, J.M.; Peres-Ueno, M.J.; de Matos, B.B.R.; Scudilio, G.M.; de Abreu, D.C.C. Diagnostic Accuracy of the Five Times Stand-to-Sit Test for the Screening of Global Muscle Weakness in Community-Dwelling Older Women. *Exp. Gerontol.* 2023, **171**, 112027. https://doi.org/10.1016/j.exger.2022.112027.
11. Furlanetto, K.C.; Correia, N.S.; Mesquita, R.; Morita, A.A.; do Amaral, D.P.; Mont'Alverne, D.G.B.; Pereira, D.M.; Pitta, F.; Dal Corso, S. Reference Values for 7 Different Protocols of Simple Functional Tests: A Multicenter Study. *Arch. Phys. Med. Rehabil.* **2022**, *103*, 20–28. https://doi.org/10.1016/J.APMR.2021.08.009.
12. Gallagher, D.; Heymsfield, S.B.; Heo, M.; Jebb, S.A.; Murgatroyd, P.R.; Sakamoto, Y. Healthy Percentage Body Fat Ranges: An Approach for Developing Guidelines Based on Body Mass Index. *Am. J. Clin. Nutr.* 2000, **72**(3), 694-701. https://doi.org/10.1093/AJCN/72.3.694.
13. Bhasin, S.; Travison, T.G.; Manini, T.M. et al. Sarcopenia Definition: The Position Statements of the Sarcopenia Definition and Outcomes Consortium. *J. Am. Geriatr. Soc.* 2020, **68**(7), 1410e8. https://doi.org/10.1111/JGS.16372.
